# The Reporting Quality Assessment of Complex Interventions' Articles in Traditional Chinese Medicine

**DOI:** 10.1155/2013/250690

**Published:** 2013-07-17

**Authors:** Meng Wu, Jingqing Hu, Biaoyan Liu

**Affiliations:** ^1^Clinical Evaluation Center, Guang'anmen Hospital, China Academy of Chinese Medicine Sciences, Beijing 100053, China; ^2^Henan College of Traditional Chinese Medicine, Zhengzhou 450008, China; ^3^China Academy of Chinese Medicine Sciences, Beijing 100700, China

## Abstract

*Objective.* To realize the current situation and problems of complex interventions' clinical trials. *Methods.* Searching at Chinese Journal Integrated Traditional and Western Medicine and Journal of Traditional Chinese Medicine from 2007 to 2012 by hand, we identified complex interventions' articles, and then we used the proposed criteria of complex interventions and CONSORT FOR TCM to evaluate. *Results.* All data is presented as counts with percentages and details in tables. *Conclusion.* Our evaluation presented that complex interventions have many defects: the selection of the intervention's components lacks rationale, complex interventions were short of fundamental researches, components' interactions were ambiguous, and the advantages of complex interventions were not mentioned. Furthermore, explanation of sample size, blind, quality control, ethical approval, and inform consent were neglected in different degrees.

## 1. Introduction

Treatments' diversity is a feature of Traditional Chinese medicine. Traditional Chinese medicine includes decoctions, acupuncture, moxibustion, and massage. More than one of the above were used at the same time, which can be defined as complex interventions. What complex interventions means? In 2000, the British Medical Research Council (MRC) had proposed the following definition: “complex interventions are built up from a number of components, which may act both independently and interdependently. The components usually include behaviors, parameters of behaviors (e.g., frequency, timing), and methods of organizing and delivering those behaviors (e.g., type(s) of practitioner, setting and location)” [1].

 Therefore, only to improve the implementation and evaluation of complex interventions can make TCM more scientific and easier to grasp. Traditional Chinese medicine and western medicine have to face the common problem. The complex interventions have been concerned in the world; several scholars have put forward some models for complex interventions' trials; the most common models are van Meijel et al. model, Bradley et al. Model [2], and the framework for the development and evaluation of complex interventions published by MRC. But the method for complex interventions' researches is still very short; only Ralph Möhler et al. [3] proposed criteria for reporting the development and evaluation of complex interventions in healthcare. 

 In order to realize the methodological problems of complex interventions' researches in TCM, we assessed the quality of complex interventions' articles published in Chinese Journal Integrated Traditional and Western Medicine, Journal of Traditional Chinese Medicine from 2007 to 2012, 12 volumes 168 issues in total. Based on the framework by MRC, an evaluation of these complex interventions' articles was made by using the proposed standards special for evaluating the complex interventions. In addition, we also used CONSORT FOR TCM [4] criteria, which are applicable to evaluating RCTs of TCM proposed by Chinese Cochrane Center, to evaluate general things of these articles. Our assessment intends to find out the problems of complex interventions' trials and inadequacies in article writing, so that we can give some suggestions to latecomers.

## 2. Methods

### 2.1. The Sources of Material

 We selected complex interventions' articles published in Chinese Journal Integrated Traditional and Western Medicine, Journal of Traditional Chinese Medicine in 2007–2012 by hand.

 Inclusion criteria: (1) a complex intervention as defined by MRC [1], (2) clinical trials.
 Exclusion criteria: (1) without control interventions, (2) articles belong to case reports or short reports.

### 2.2. Methods and Content

#### 2.2.1. Methods

 Two trained evaluators independently evaluated all articles based on the criteria, checked jointly, and then resolved disagreements by consensus. 

#### 2.2.2. Evaluation of Complex Intervention Articles

1. The general things of articles (see Tables 1 and 2).
2. The proposed topics for reporting the complex interventions (see Table 3).
3. The CONSORT FOR TCM criteria (see Table 4).

#### 2.2.3. Statistical Methods

No formal statistical tests were used; all data is presented as counts with percentages.

## 3. Results

 In 2007−2012, Chinese Journal Integrated Traditional and Western Medicine and Journal of Traditional Chinese Medicine have published 801, 540 clinical trials' articles, respectively, 244, 143 of them belong to complex interventions. Finally, 354 articles found and most studies on gynecology and reproductive system diseases (13%), followed by bone and joint or motion sickness, Tumor, digestive diseases, endocrine diseases. 89.3% of the trials only have one participating center, only 118 (33.3%) trails funded by national, provincial, ministry, or municipal funding for the data in detail, see Tables 1 and 2.

 Details on the evaluation based on the purposed content are presented in Table 3 and evaluation based on the CONSORT FOR TCM is in Table 4. 

## 4. Discussion

 According to the result of our assessment, we can realize the current situation and shortages of complex interventions in TCM as follows.

### 4.1. The Proposed Content Special for Complex Interventions Trials

#### 4.1.1. The Introduction of Background Which Leads to the Complex Interventions

 The complex interventions are widely used, but are not meant for abuse of them, Professor Jialiang Wang thinks the clinical application of complex interventions requires certain conditions. It is usually applied to serious chronic diseases or epidemic diseases which still cannot be well treated. All these diseases were complicated and cannot be treated with a single measure [5]. So we think researchers should make a reasonable explanation of the target disease, including the shortcomings of current treatment methods and the disadvantages of single components which can support us to use complex interventions. The evaluation showed that many articles deficient in this information, even not mentioned.

#### 4.1.2. The Type of Trial's Objective

 According to research methods and designs, all studies can be divided into two major types, namely, exploratory studies and confirmatory studies. The latter has a more strong ability of confirmation than the former to evaluate the therapeutic efficiency [6].

 Most included articles intended to observe the clinical efficacy of the treatments, so they cannot provide solid evidences. What is more, a lot of researchers have not clearly described what the effects should have. Only a small part of articles belong to confirmatory studies.

#### 4.1.3. Selection of the Intervention's Components

 Complex interventions are combinations of several components. How to select components for complex interventions is a primary problem to solve. Following the framework by MRC, the first step is identifying existing evidence [7]. Evidence for complex interventions may come from systematic reviews, animal studies, clinical researches, or accumulation of personal clinical experiences. Making all interventions based on best evidence is not always realistic clinical medicine will be more practical [5], so plenty of effective interventions; get from accumulations of experience and deepening researches. At least, the selection of components cannot be fabricated.

 As results of the evaluation presented, researchers have not paid attention to select components scientifically many articles; are without mention. Evidence selection was given by few articles, which mostly came from animal studies or clinical researches. In addition, the studies on Chinese medicine decoctions are still in the stage of taking apart prescriptions, that is, study on herbs, respectively, rather than regarding them as a whole. 

#### 4.1.4. Rationale for Theoretical Basis of Combining the Complex Interventions' Program

 The interactions between different components are complex, which will make a difference in complex interventions' efficacy. Therefore, we think that designing a complex intervention also should be based on strong evidence. Wherever possible, evidence should be combined from different sources that do not share the same weaknesses [8]. The evaluation revealed that most complex interventions' trials lack sufficient and scientific evidence; that is to say, what theories support us to combine this rather than those components is not clear. Researchers should develop the complex interventions' program following the evidence-based science.

#### 4.1.5. The Type of Complex Interventions' Designs

 As the efficacious method to prevent selection biases, randomization is always the first to be considered. 88.1% of these articles claimed that their trials were randomized controlled trials, but not all of them have stated the method of random generation. Whether randomizations were true or not cannot be speculated. 

 Although RCT is good, it also has some limitations in the process of implementation. RCT should based on reasonable hypothesis and good generalizability, which can make it more valuable [9]. Sometimes, randomization may be unnecessary and other designs preferable [7, 10]. More and more practical randomized controlled trials (PRCTs) were applied to clinical complex interventions trials, no longer limited in explanatory randomized controlled trial (ERCT) [11]. The new guidelines released by MRC in 2008 provided several designs for complex interventions evaluation, which including individually randomized trials, cluster randomized trials, stepped wedge designs, preference trials, and randomized consent designs, N of 1 designs [7]. Researchers should choose a more appropriate one instead of using ERCT all the time.

#### 4.1.6. The Combination Type of Intervention's Components and Pilot Study

 The combinations of complex interventions in these articles involved various formations. In order to know the form of combinations, we classified them roughly. “Chinese medicine” included decoctions and Chinese patent drugs; “Western medicine” included western medicines and conventional or basic therapies; “nondrug treatments” included acupuncture and moxibustion, massage, radiotherapy, surgery, therapeutic equipments (such as laser, and ultrasound), and gymnastics. Trials which combined Traditional Chinese medicine and Western Medicine were more common in the articles reviewed. Evaluations are often undermined by problems of acceptability, compliance, delivery of the intervention, recruitment and retention, and smaller than expected effect sizes that could have been predicted by thorough piloting [7, 12]. But none of the included articles has pilot study.

#### 4.1.7. Control Interventions and Evaluating Outcomes

 Positive drugs are more often selected as control interventions. Placebo controls were not common, which may be caused by potential ethical issues.

 The results of complex interventions' trials would be more various than single intervention. Using different kinds of methods to evaluate the results will be more objective and comprehensive. It is important also to consider which sources of variation in outcomes matter and to plan appropriate subgroup analyses [7].

#### 4.1.8. Categorizing Interactions between Different Components

 Elaboration of interactions between components is very short in included articles. Three kinds of interactions may make the efficacy better: some similar components combined for enhancing the overall efficacy; components complemented each other; one component reduced the side effects of another. But none of the articles claimed conclusion that efficiency was reduced by using the complex interventions; it means all the researchers considered that complex interventions were better than a single intervention. Most complex interventions are still in the stage of observing the clinical efficacy, and studies of interactions between components are very short; it is essential to do further confirmatory studies in future, which will also become a focus of medicine researches in future.

#### 4.1.9. The Advantages of Using Complex Interventions

 Although vast majority of researchers have claimed that their complex interventions were better than single intervention, most of them only mentioned “better efficacy” lightly, no specific and detailed explanation of the advantages. Furthermore, plenty of articles limited to the comparison of clinical efficacy between different interventions and ignored the comparisons of convenience and economics' aspect which would make the evaluation more comprehensive.

### 4.2. Overview of General Things and Evaluation Results Based on CONSORT FOR TCM

 Besides the evaluation special for complex interventions, we have assessed these articles based on CONSORT FOR TCM criteria. Then we compared our result to an earlier similar study which evaluated RCTs published on Chinese Journal Integrated Traditional and Western Medicine in 1999–2004.

 The results of our evaluation presented that few researchers used the diagnostic criteria and syndromes of TCM (42.7%). So we can speculate that some researches only connected one disease with certain medicine(s) without using syndrome differentiation of TCM. 

 About selecting outcome measures, intermediate indicators were more common than endpoint which may be due to its shorter time for observation. The fact that less use of syndrome indicators (24.9%) revealed that many trials are still in the stage of “diseases without syndrome.” The indicators except relating to efficacy, such as economic indicators, were rarely used. 

 The most significant problem of giving interventions' details is the unclear description of the quality control of Chinese medicine decoctions, including the metropolis, batch number, and concocting method, which has greatly increased the biases of Chinese medicine in the process of using and the boiling. In order to solve the problem, some researches use granules of Chinese medicines instead of herb pieces to reduce the biases. But Weixiong Liang thinks that Chinese medicine granules also have two major shortages. Firstly, they only have production standards of Chinese medicine granules without national standards of quality control. Secondly, in the process of boiling, herb pieces would interact with each other, but granules lack the link [13]. The two points above will be the main bottleneck of Chinese medicine granules.

 Although most articles have explained the results, not all of them explained both on statistics and clinical areas. Deficiency of the explanation based on TCM theory is a common issue of the clinical trials which selected Chinese patent drugs or nondrugs as the interventions. Furthermore, there are several problems with complex interventions' trials just as general RCTs, including quality control, and informed consent and ethical approval. A great number of articles have not listed the deficiencies or potential biases of their trials and have not mentioned the acknowledgments or conflicts.

 Comparing our results with a similar assessment made by Mao et al. who used Consort statement to evaluate RCTs published in the Chinese Journal of Integrated Traditional and Western medicine in 1999–2004 [14], some large improvements have been presented: the reporting of exclusion criteria increased from 28.42% to 71.8%, statistical methods (77.95% to 98%), statistic in detail (33.3% to 91.2%), adverse events (34.71% to 55.9%), informed consent (0.24% to 38.7%), follow-up records (11.42% to 29.1%), and syndromes differentiation of TCM (12.66% to 42.7%). The sample size determination, blinding, ethical approval, flow chart, and analysis of potential biases all improved very little. Therefore, the quality of RCTs still has a large room for improvement.

Zhan et al. have evaluated the RCTs published on several Chinese medicine journals from 1976 to 1996 [15], however, their evaluation presented that non-single intervention trails only accounted for 19%. These years, the quantity of nonsingle intervention trials has increased, which accounted for 1/3 each year more or less. But the publication biases still exist; 352 included articles stated positive results, which may lead readers to draw some wrong conclusions about treatment efficacies. 

 Compared to the results of a similar foreign assessment, we can see that there were large gaps between them. Mills et al. investigated 253 articles published in the BMJ, JAMA, Lancet Archives, and Annals about the quality of randomization [16]. The results showed that sequence generation is 80.2%, allocation concealment is 48.2%, description of the blinding method is 81.4%, and sample size is 82.6%. Flow charts were also more common. All of the above were better than RCTs in TCM.

## 5. Summary

 As the results of our quality assessment showed, complex interventions articles in TCM need to be improved urgently. The descriptions of theories or primary researches supporting the complex interventions are insufficient and unreliable; great majority of researchers designed their complex interventions depending on experiences. Explanations of interactions between different components were inadequate; the researchers have not explained the relationships between them. All these are important points to a complex interventions' trial. Most interventions of included articles mixed with western medicines, Chinese patent drugs, injection therapies, and so forth, which perhaps reduced the proportions in some items of TCM. 

 Also our evaluation has some shortages; the proposed criteria for complex interventions are immature, perhaps some key contents are missing. 

 With the development of evidence-based medicine in recent years, some aspects of RCTs are already better than before. As more and more people pay attention to the evaluation of complex interventions, we are convinced that someone will propose a better criteria for evaluating the complex interventions in the near future, and the complex interventions trials will be improved.

## Figures and Tables

**Table 1:** Data sources.

	2007	2008	2009	2010	2011	2012
Chinese Journal Integrated Traditional and Western Medicine						
Clinical trials' article	181	146	131	99	101	143
The complex interventions' articles belong to clinical trials	78 (43.1%)	44 (30.1%)	39 (29.8%)	24 (24.2%)	31 (30.7%)	28 (19.6%)

Journal of Traditional Chinese Medicine						
Clinical trials' article	63	68	58	60	155	136
The complex interventions' articles belong to clinical trials	19 (30.2%)	15 (22.1%)	14 (24.1%)	19 (31.7%)	28 (18.1%)	48 (35.3%)

**Table 2:** The data of included articles in general.

Item	Sub-item	Number of reported (%)
The top five types of diseases that articles involve are	Gynecology and reproductive system diseases	46 (13.0%)
	Bone and joint or motion sickness	41 (11.6%)
	Tumor	39 (11.0%)
	Digestive diseases	37 (10.4%)
	Endocrine diseases	32 (9.0%)

Funding	National	41 (11.6%)
	Provincial, ministry, or municipal	77 (21.8%)
	Others	10 (2.8%)
	No explanations	226 (63.8%)

The department of first author	Colleges, universities, and affiliated hospital	188 (53.1%)
	Other hospitals at all levels	139 (39.3%)
	Research institutions	27 (7.6%)

The number of participating centers	1	316 (89.3%)
	2–5	32 (9.0%)
	6+	6 (1.7%)

Other information	Informed Consent	137 (38.7%)
	Ethical approval	9 (2.5%)
	Acknowledgments	3 (0.8%)

**Table 3:** The results of using the proposed criteria for evaluation.

Item	No.	Subitem	Number (%)
The introduction of background which lead to the complex interventions	1	Introduction of target disease	214 (60.5%)
		Limitations of using single interventions	145 (41.0%)
		Not mentioned	93 (26.3%)

The type of objectives	2	Main purpose of observing clinical efficacy	324 (91.5%)
		Demonstrate results of former research(es)	30 (8.5%)

Rationale for the selection of the intervention's components	3	Depend on systematic review results of clinical trials	1 (0.3%)
		Based on former clinical trials	99 (28.0%)
		The results of animal studies	112 (31.6%)
		Experts consensus	5 (1.4%)
		Personal clinical experiences	29 (8.2%)
		Not mentioned	108 (30.5%)

Description the theoretical basis of complex interventions' program	4	Depend on systematic review results of clinical trials	0 (0.0%)
		Based on former clinical trials	25 (7.1%)
		The results of animal studies	0 (0.0%)
		Experts consensus	1 (0.3%)
		Personal clinical experiences	11 (3.1%)
		Not mentioned	317 (89.5%)

Pilot study	5	Is there pilot study or not?	0 (0.0%)

Sample size	6	<50	32 (9.0%)
		50–99	193 (54.5%)
		100–299	118 (33.3%)
		≥300	11 (3.1%)

The type of design	7	Randomized controlled trials	312 (88.1%)
		(i) Randomization	225 (63.6%)
		(ii) Quasi-randomization	49 (13.8%)
		(iii) Randomized, no method stated	38 (10.7%)
		Cohort study	8 (2.3%)
		Crossover design	1 (0.3%)
		Case-control study	1 (0.3%)
		Non-randomized concurrent controlled trials	32 (9.0%)

The categories of combinations between intervention's components	8	Chinese medicine alone	113 (31.9%)
		(i) Chinese medicines with different formulations	22 (6.2%)
		(ii) Chinese medicines + non-drug	67 (18.9%)
		(iii) Difference kinds of non-drug TCM	24 (6.8%)
		Western medicine alone	0 (0.0%)
		Chinese plus western medicine	241 (68.1%)
		(i) Chinese medicines + western medicines/basic treatments	207 (58.5%)
		(ii) Western medicines + non-drug	20 (5.6%)
		(iii) Chinese medicines + western medicines + non-drug	14 (4.0%)

Control interventions	9	A single component as effective control	156 (44.1%)
		Several components combined as a effective control	185 (52.3%)
		Placebo control	12 (3.4%)
		Blank control	1 (0.3%)

Indicators for evaluating the outcomes	10	One indicator	116 (32.8%)
		Multiple indicators for comprehensive evaluation	238 (67.2%)

Elaborating interactions between different components	11	Interactions lead to better efficacy	118 (33.3%)
		(i) Some similar components combined for enhancing the overall efficacy	60 (16.9%)
		(ii) Components complemented each other to enhance the efficacy	27 (7.6%)
		(iii) Improved by reducing side effects	31 (8.8%)
		Interactions lead to worse efficacy	0 (0.0%)
		(i) Efficacy reduced without side effect	0 (0.0%)
		(ii) Some side effects generated	0 (0.0%)
		Not mentioned	236 (66.7%)

The advantages of using complex interventions' program	12	More effective	297 (83.9%)
		Shorten the treatment time	24 (7.6%)
		Fewer side effects (security)	96 (27.1%)
		Easier to implement	19 (5.4%)
		Less cost of treatment	18 (5.1%)
		Not mentioned	36 (10.2%)

**Table 4:** The results of assessment based on CONSORT FOR TCM.

Paper section and topic	Item	Description	Number (%)
Title and abstract	1	Is there abstract or not?	269 (76.0%)
Introduction			
Background	2	A brief statement of reasons for trials and medicines	261 (73.7%)
Objectives	3	Specific objectives or hypotheses	237 (66.9%)
Methods			
Participants	4	Diagnostic criteria of Western medicine	299 (84.5%)
		Using the diagnostic criteria and Syndromes of TCM	151 (42.7%)
		Inclusion criteria	268 (75.7%)
		Exclusion criteria	254 (71.8%)
		The settings and location where the data collected	301 (85.0%)
Interventions	5	Details on interventions of each group (such as dosage, metropolis, concocting method, and batch number)	161 (45.5%)
		Strategy of quality control	5 (1.4%)
Outcomes	6	Clearly defined outcome measures and when applicable	256 (72.3%)
		Using the syndrome indicators to evaluate the outcome	88 (24.9%)
Sample size	7	How sample size was determined	4 (1.1%)
Randomization			
(i) Sequence generation	8	Method used to generate the random allocation sequence	260 (73.4%)
(ii) Allocation concealment	9	Method used to implement the random allocation sequence	29 (8.2%)
(iii) Implementation	10	Who generated the allocation sequence, who enrolled participants, and who assigned participants to their groups?	12 (3.4%)
(iv) Blinding	11	Single blinding	8 (2.3%)
		Double blinding	13 (3.7%)
		Not mentioned	333 (94.1%)
Statistical methods	12	Description of statistical methods	347 (98.0%)
Results			
Participant flow	13	Flow of participants' changes in each stage	90 (25.4%)
		A diagram of flow	0 (0.0%)
		Compliance	15 (4.2%)
Recruitment	14	The method for collection	6 (1.7%)
		Follow-up record	103 (29.1%)
Baseline data	15	Baseline demographic and clinical characteristics of each group	68 (19.2%)
Numbers analyzed	16	Whether the analysis was by ‘‘intention-to-treat”	7 (2.0%)
Outcomes and estimation	17	Positive results	352 (99.4%)
		Negative results	0 (0.0%)
		Equivalent results	2 (0.6%)
		The precise of data	323 (91.2%)
		Give the confidence interval and value of “*P*”	40 (11.3%)
Ancillary analyses	18	Results of any other analyses performed, including Subgroup analyses and adjusted analyses, distinguishing prespecified from exploratory	5 (1.4%)
Harms	19	All important harms or unintended effects in each group	198 (55.9%)
Discussion			
Interpretation	20	Interpretation of the results	353 (99.7%)
		Explaining the significance in statistics and treatment	255 (72.0%)
		Explain the results with theories of TCM	218 (61.6%)
		Potential bias	28 (7.9%)
Generalizability	21	Generalizability (external validity) of the results	86 (24.3%)
Overall evidence	22	Description of the interest conflict between researchers and participants	0 (0.0%)

Note: items in Tables 3 and 4 are calculated one by one; few items may cross with each other, so we counted them more than one.
